# Teachers’ visual processing of children’s off-task behaviors in class: A comparison between teachers and student teachers

**DOI:** 10.1371/journal.pone.0259410

**Published:** 2021-11-03

**Authors:** Hirofumi Shinoda, Tsuyoshi Yamamoto, Kyoko Imai-Matsumura

**Affiliations:** 1 Graduate School of Education, Bukkyo University, Kita-ku, Kyoto, Japan; 2 Wako high school, Wako, Saitama, Japan; Kyoto University, JAPAN

## Abstract

As teachers are responsible for responding instantaneously to students’ statements and actions, the progress of the class, and their teaching purpose, they need to be able to engage in responsive teaching. Teachers obtain information about students’ learning by observing them in the classroom, and subsequently make instructional decisions based on this information. Teachers need to be sensitive to student behaviors and respond accordingly, because there are students who follow the teacher’s instructions and those who do not in every classroom. Skilled teachers may distribute their gaze over the entire class and discover off-task behaviors. So how does a teacher’s visual processing and noticing ability develop? It is important to clarify this process for both experienced teachers and student teachers. Therefore, the purpose of this study was to investigate whether there is a difference in visual processing and the ability to notice off-task behaviors in class between teachers and student teachers through gaze analysis. Using an eye tracking device, 76 teachers and 147 student teachers were asked to watch a video, and gaze measurements were collected. In the video, students exhibiting off-task behaviors in class were prompted by their classroom teacher to participate in the lesson. After the video, the participants were asked if they could identify the students who had displayed off-task behaviors and whom the teachers had warned. The results showed that teachers gazed at students engaging in off-task behaviors in class more often and noticed them at a higher rate than student teachers did. These results may be attributed to differences in the experiences of visual processing of relevant information in the classroom between teachers and student teachers. Thus, the findings on teachers’ visual processing by direct measurement of gaze will be able to contribute to teachers’ development.

## Introduction

### The need for responsive teaching

To be able to engage in their main activity—teaching—teachers must be skilled in managing complex classroom environments [1]. Teachers choose what they will pay attention and respond to, while simultaneously thinking about relationships between students’ remarks and higher-order principles of teaching and learning [2]. It is thought that effective teaching is created by adapting in response to the environment one faces, including the classroom environment and its diversity [3]. According to Seidel and Stürmer [4], “even in short sequences of classroom teaching, a myriad of teaching and learning acts occur” (p. 742). Moreover, teachers often encounter situations with high variability; thus, they must adapt their teaching style to the environment and students [3]. This ability to adapt to what is occurring in the classroom in the moment is known as responsive teaching. As teachers are required to respond instantaneously to students’ statements and actions, the progress of the class, and their teaching purpose, they need to be able to engage in responsive teaching [5]. As mentioned above [2–5], responsive teaching requires the ability to see the behavior of children during class and respond instantaneously considering the development and purpose of the class. Although teachers should have the abilities necessary for responsive teaching, empirical research on the structure and development of teaching skills [4] is limited.

### Noticing and interpreting during responsive educational activities

Responsive teaching depends on teachers’ abilities to notice and interpret interactions between themselves and students, which is vital for improving teaching skills [2]. Furthermore, responsive teaching requires teachers to focus not on predetermined goals and objectives, but on what is happening in front of them in the moment. Thus, responsive teachers build relationships directly from what students are doing and discussing [6]. Moreover, teachers obtain information about students’ learning by observing them in the classroom and then making instructional decisions based on this information [7]. Such decisions are also influenced by aspects of a teacher’s professional vision, such as awareness and interpretation [2, 8]. Furthermore, classroom management, as the foundation of classroom learning, is strongly related to teachers’ in-class perceptions and interpretations [9]. In this context, “noticing” is whether teachers attend to relevant events in the classroom, and teachers’ awareness of situational complexity is an important element of teaching expertise [1, 4]. Teachers need to be sensitive to student behaviors and respond accordingly, because there are students who follow the teacher’s instructions and those who do not in every classroom.

### Teachers’ visual processing

Teachers continuously need to monitor the classroom environment to understand cues and events in children’s behavioral changes, and this integrated cognitive processing is provided by their visual and perceptual abilities [10]. According to the teacher’s decision-making model by Westerman [11], experienced teachers implement their teaching plans and behavior based on their awareness of, and monitoring for, children’s cues. In class, novice teachers tend to pay attention to cues from individual children, while experienced teachers pay attention to individuals as well as groups [12]. Furthermore, Carter, Cushing, Sabers, Stein, and Berliner [13] investigated how expert teachers, novice teachers, and applicants who were interested in teaching but had no training or experience perceived visual classroom stimuli. They reported that applicants assigned equivalent informational value to all visual stimuli and characterized static features of the classroom environment in detail; however, experts paid attention to students’ work state and form and understood the connection between student behaviors and the classroom situation [13]. Meanwhile Swanson, O’Connor, and Cooney [14] presented a short scene on classroom discipline problems to expert and novice teachers and found that expert teachers shift their attention to clarify a problem because they have a well-established strategy to solve discipline problems in the classroom.

According to the findings of these previous studies, when observing visual classroom stimuli, teachers preferentially perceive and select information that is meaningful for educational activities [15] and discard information that is irrelevant. Thus, the development of a teacher’s ability to engage in responsive teaching successfully is reflected in visual processing. In addition to these previous studies, studies have been conducted in recent years to directly evaluate the teacher’s visual processing of the behavior of children in order to clarify the teacher’s visual attention [7, 10, 16–18]. However, the number of such studies remains small.

### Relationships between expertise and visual processing

Visual processing can be measured by tracking eye movements [19]. Eye-tracking is a key element in human social cognition research [20] and helps reveal subtle cognitive processes that are otherwise difficult to observe [21]. Moreover, recording eye movements by eye tracking is useful for studying human behavior that cannot be intuitively associated with visual processing [22]. Further, results from eye-tracking studies show that visual processing is influenced by expertise; as task knowledge increases, an individual’s gaze tends to stop more quickly and linger longer on task-related information [23]. Research using eye tracking has shown that compared to novices, experts focus their eye gaze on the target object and subsequently develop the related visual and cognitive processing. The domains/fields demonstrating this effect are diverse and include flight simulation [24], programming [25], chess [26, 27], sports [28–30], art [31], medicine, biology [32–34], and mathematics [35, 36].

### Relationship between expertise and visual processing in teachers

Recent research [7, 10, 16–18] has shown that eye-tracking is an effective measure of teachers’ visual attention. There are differences in the number of eye fixations and eye fixation durations of experience teachers for target people or objects, compared to inexperienced teachers and student teachers [7, 10, 16–18]. According to Van den Bogert, van Bruggen, Kostons, and Jochems [16], when watching videos of students’ off-task behaviors during class, student teachers tended to ignore other classroom areas as they focused on a single student. However, experienced teachers kept their eyes on the off-task behavior, while also continuing to observe the rest of the classroom. Wolff, Jarodzka, van den Bogert, and Boshuizen [10] showed four videos that included students’ off-task behaviors to both experienced and student teachers and measured their eye movements. The findings indicated that it was a professional skill to systematically visually survey the environment for valuable cues. McIntyre, Mainhard, and Klassen [17] investigated differences in gaze between expert and novice teachers in the UK and Hong Kong and found that experts in both countries showed longer gaze durations for students, indicating that experts were more student-centered than novices. Cortina, Miller, McKenzie, and Epstein [18] compared the distribution of gazes at students between experienced and novice teachers and found that experienced teachers could consistently and continuously observe a classroom, even when assisting one particular student. Yamamoto and Imai-Matsumura [7] provided another example of eye-tracking research by measuring teachers’ gaze toward students. They found that teachers who noticed students’ off-task behaviors fixated on those students more frequently than teachers who did not notice such behaviors and that there was no difference in the duration of each fixation. Furthermore, they found no difference in teaching experience between teachers who were aware and those who were unaware of off-task behaviors.

These previous studies have revealed teachers’ visual processing of children’s behavior by tracking eye movements, which is a quantitative indicator. In more subtle studies, interest in the gaze object is related to the frequency of gazes (the number of fixations) [7], and the acquisition of information from the gaze object is related to the time (fixation duration) [37, 38]. During class, students are distracted by unrelated issues approximately 10–50% of the time [39–41]. Given this situation, it is important to study teachers’ ability to notice off-task behaviors. However, studies that analyze teachers’ gaze are limited. In a review of gaze analysis for teachers, Beach and McConnel [42] point out the small sample sizes of previous studies. Additionally, few studies focus on teachers’ instructions and students’ off-task behavior in response to those instructions or analyze teachers’ gaze distribution toward students engaging in off-task behavior. A counter-intuitive finding from a previous study conducted by Yamamoto and Imai-Matsumura [7], that among teachers, years of teaching experience were not related to visual processing or noticing abilities, warrants additional attention. This study only considers years of teacher experience and shows that visual processing and noticing abilities vary widely among teachers. Most gaze analysis studies of teachers indicate a link between experience / specialty and perception / interpretation of classroom situations [10, 16–18]. Teachers strengthen the skills necessary for effective education through on-site experience, and teachers’ perception, understanding, and interpretation of classroom situations are greatly influenced by the expertise accumulated from their experience [43]. If years of teaching experience are not related to visual processing and awareness abilities, what is? Does the teacher’s experience itself involve the teacher’s visual processing and awareness abilities? It is an important issue for teacher education.

### Research questions and study purpose

In Japan, students at teacher training schools receive a teaching license upon graduation from university after receiving a certain level of education and training. After that, they become teachers if they pass the employment examination of the local board of education. Given the level of training teachers receive, in the current study we asked the following question: Do the visual processing and student-awareness abilities of students who have only had teaching practice differ from those of experienced in-service teachers (employed classroom teachers)? If there are no differences between the two groups, are differences instead due to teachers’ innate temperament? In this study, we focused on differences in visual processing and the ability to notice children with off-task behavior between students taking university-level teacher training courses (hereafter called “student teachers”) and in-service teachers who are actually teaching in the classroom (hereafter called “teachers”). Given previous findings, we expected that teachers would notice and focus on important events in the classroom more than student teachers would. Moreover, it is worth noting that both groups’ visual processing can be directly measured by eye movements, via eye-tracking.

The purpose of this study was to determine whether there are differences in the visual processing and the ability to notice students’ off-task behaviors in class between teachers and student teachers. The teachers and student teachers were asked to watch a video recording of the students’ behavior in class, from the perspective of the teacher teaching the class, and their gazes while watching were examined. In our video, we used a scene in which an elementary school teacher noticed children who were not responding to instructions during the class and warned the children. Based on findings from previous studies, we set the following hypotheses:

1. Teachers direct their gaze to children who show off-task behaviors in class more often and for longer than student teachers do because experienced teachers consistently observe children in class and repeatedly gaze at valuable cues [10, 16–18].
2. Teachers notice children who show off-task behaviors in the classroom more than student teachers do, because teachers preferentially perceive information meaningful for classroom management [15].

To explore these hypotheses, we examined the differences in teacher and student teachers’ gaze on children’s behavior.

## Materials and methods

### Participants

A total of 223 people participated in this study, including 76 elementary school teachers (32 men, 44 women; mean age: 39.96 ± 9.46 years; mean years of teaching experience: 15.91 ± 9.08 years, minimum age: 23 years, maximum age: 61 years) and 147 student teachers (66 men, 81 women; mean age: 21.64 ± 1.88 years; minimum age: 18 years; maximum age: 27 years). The in-service teacher participants were not limited to experienced teachers and included less experienced teachers. The student teachers were students who did not have a teacher’s license. Participants were provided a verbal and written explanation of the study, and written informed consent was obtained from them. For the children in the stimulus video, we obtained their parents’ permission to use the video. The present research was approved by the Bukkyo University Human Research Ethics Review Committee and conducted in compliance with the principles stated in the Declaration of Helsinki.

### Stimulus video

A video recording with audio of a third-grade class in a Japanese elementary school was presented to the participants for 55 seconds. The video was taken from the left front of the classroom by a fixed video camera and showed 20 children in total. In the video, the teacher was asking questions to the children, and the children were thinking and trying to answer by raising their hands. However, there were two children (target children) who were writing in their notebooks with their heads down and not responding to the teacher’s questions (off-task behavior), so the teacher warned these children that this was not the time to write and encouraged them to participate in the class. The video was filmed from the point of view of the teacher; thus, the teacher was not shown in the video, although his voice was heard.

### Procedures

Participant gaze was recorded while watching the video, using the eye-tracker T60 (sampling rate at 60 Hz) made by Tobii Technology in Stockholm, Sweden. Monitor resolution was 1024 × 768 pixels. Gaze measurement was conducted individually for each participant. The participants were seated about 60 cm from the eye-tracker, and the height was adjusted so that the eye position was at the center of the screen for each participant. The eye-tracker was calibrated by 5 points. Subsequently, the participants were told that the video was of a third-grade mathematics lesson at an elementary school, and they were asked to observe the video carefully. After these instructions, a “+” sign was posted at the center of the monitor for 3 seconds, and the video began after the participant’s gaze was fixed. The video lasted 55 seconds, during which the participant’s gaze was measured. Since the off-task behavior occurred in the latter 30 seconds of the total 55 seconds. We analyzed the numbers of fixations and fixation duration stays 25 seconds before the off-task behavior occurred (first-half 25 seconds) and 25 seconds after the off-task behavior occurred (second-half 25 seconds).

After viewing the video, to determine whether they had noticed the correct target children, researchers asked participants the following question: “Some children were warned by the classroom teacher in the video. Do you know which children?” For participants who answered “no,” the experiment was terminated. If the participants answered “yes,” they were shown a picture of the whole classroom on the screen and asked, “Which children?” And then they were examined to see if they matched the target children.

### Analysis

For the analysis, we set the target children in the video as our area of interest (AOI). AOI was defined by tracing the outline of the target children using the Tobii Studio AOI tool. Then, eye fixation duration and the number of fixations on the AOI were measured. In this study, we defined fixation as a gaze resting for 1/60 second within a radius of 35 pixels.

To compare the gaze data of student teachers and teachers with respect to the gaze to the target children in the images before and after the off-task behavior, a two-factor repeated measures ANOVA was performed for the group (teacher / student) × 2 videos (first half / second half). In addition, the same analysis was performed on the gazes to areas other than the target children. Furthermore, chi-square tests were used to investigate potential differences between noticing children’s off-task behavior and accuracy in identifying the target children. Statistical analysis was performed using SPSS for Windows, version 27 (IBM). The significance level was set at 5%.

## Results

### Teachers’ gaze toward students

**Fig 1:**
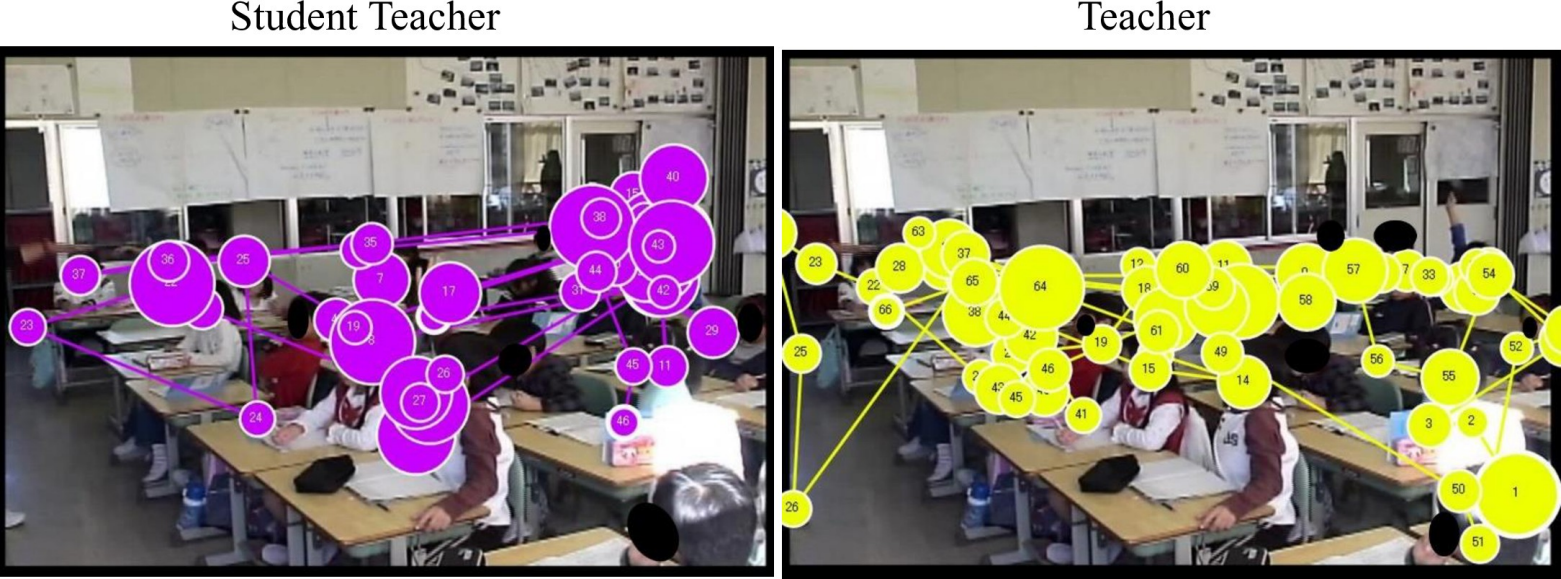
Examples of the participants’ gazes. Left indicates that of a teacher; right indicates that of a student teacher.

Fig 1 shows the eye movements of one teacher (left) and one student teacher (right) during the second-half 25 seconds when the classroom teacher warned the target children in the video. The point of fixation is a circle, and the trajectory of fixation is indicated by a straight line. The longer the fixation duration, the larger the circle becomes. Based on the individual data, the numbers of fixations on the AOI during the first-half 25 seconds and second-half 25 seconds were examined, as were fixation duration and fixation duration per fixation on the AOI during the first-half 25 seconds and second-half 25 seconds.

### Gaze to the target children

**Fig 2:**
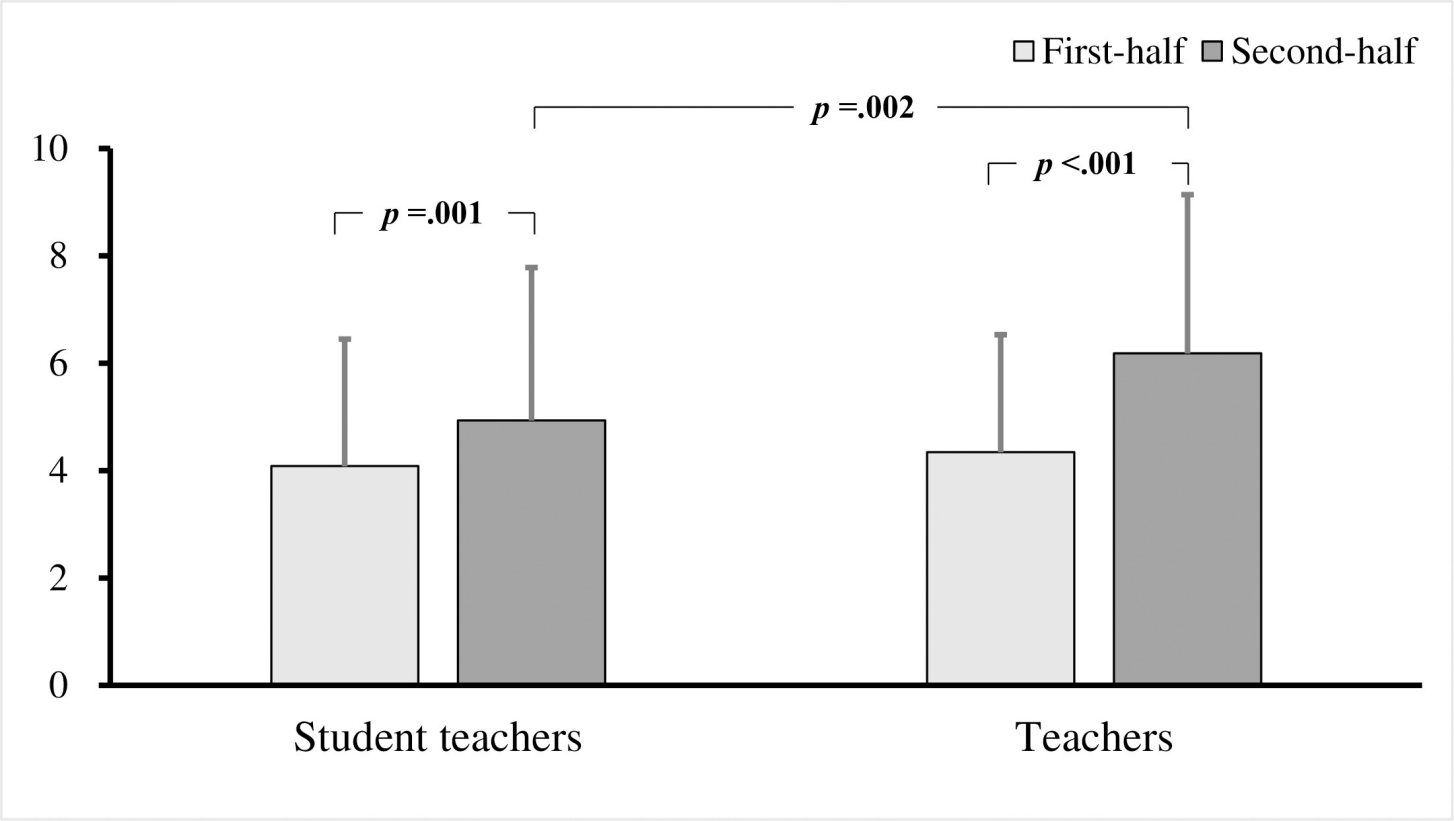
Number of fixations on target children in student teachers and teachers. Bars show the mean across all comparisons (with standard deviation bars).

**Fig 3:**
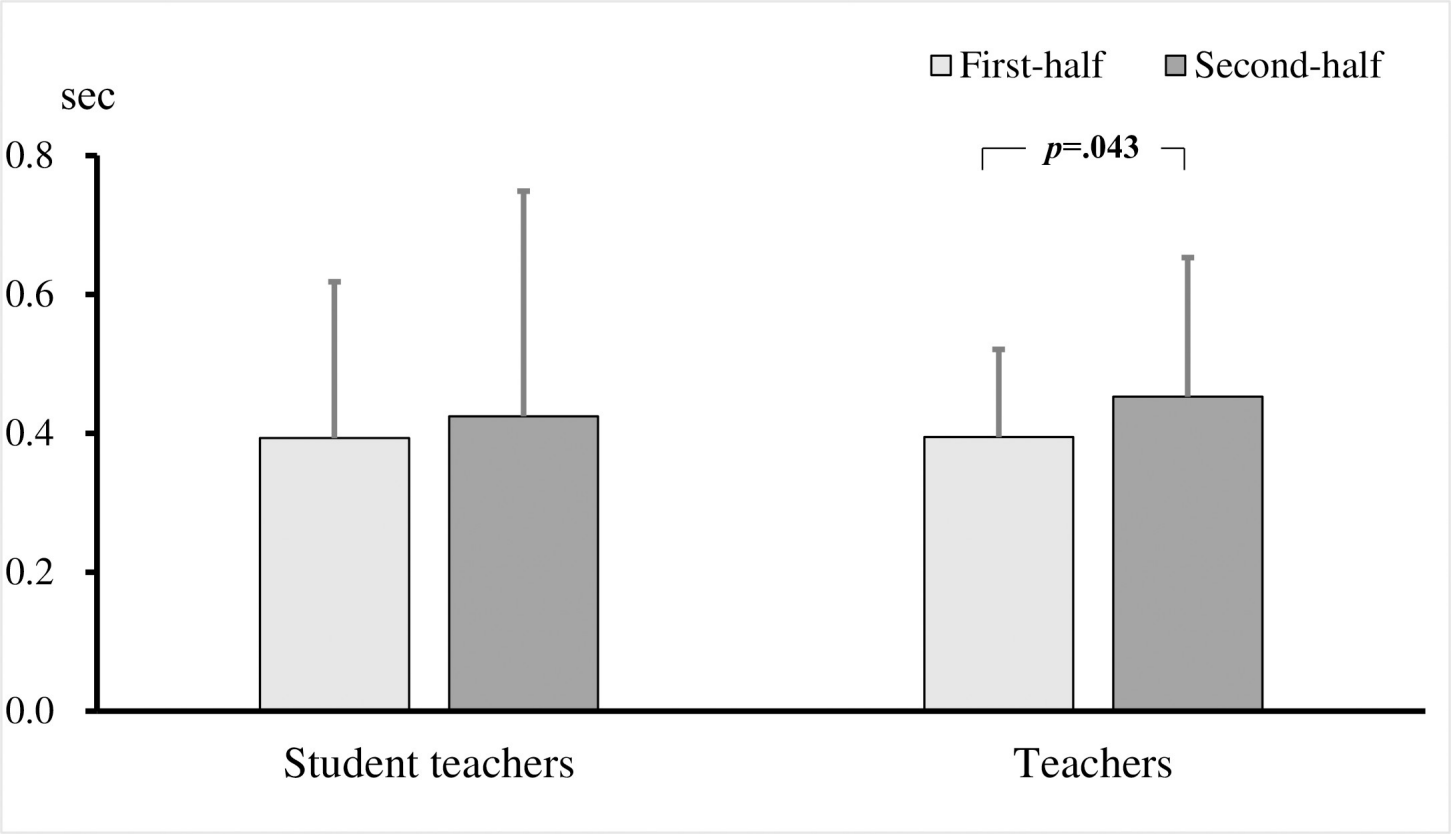
Fixation duration per fixation on target children in student teachers and teachers. Bars show the mean across all comparisons (with standard deviation bars).

To examine differences between teachers and student teachers, a two-factor repeated measures ANOVA was performed for the group (teacher / student) × 2 videos (first half / second half) on the number of fixations, fixation duration, and fixation duration per fixation. As a result, the interaction effects were significant in the number of fixations (*F* (1, 221) = 5.352, *p* = .022, *η*^2^ = .02; Fig 2). The results of the simple main effect test revealed that the number of fixations at the target child increased significantly in the second half of both the student teachers and the teachers (student teachers: *p* = .001; teachers: *p* < .001), but the number of fixations the teachers stayed in the target children in the second half was significantly higher than that of the student teachers (*p* = .002). No interaction effects were observed with respect to the fixation duration (*F* (1, 221) = 3.763, *p* = .054) and fixation duration per fixation (*F* (1, 221) = .693, *p* = .406; Fig 3). Thus, it is clear that teachers gazed more frequently toward the target children than did the student teachers.

### Gaze to areas other than the target children

**Fig 4:**
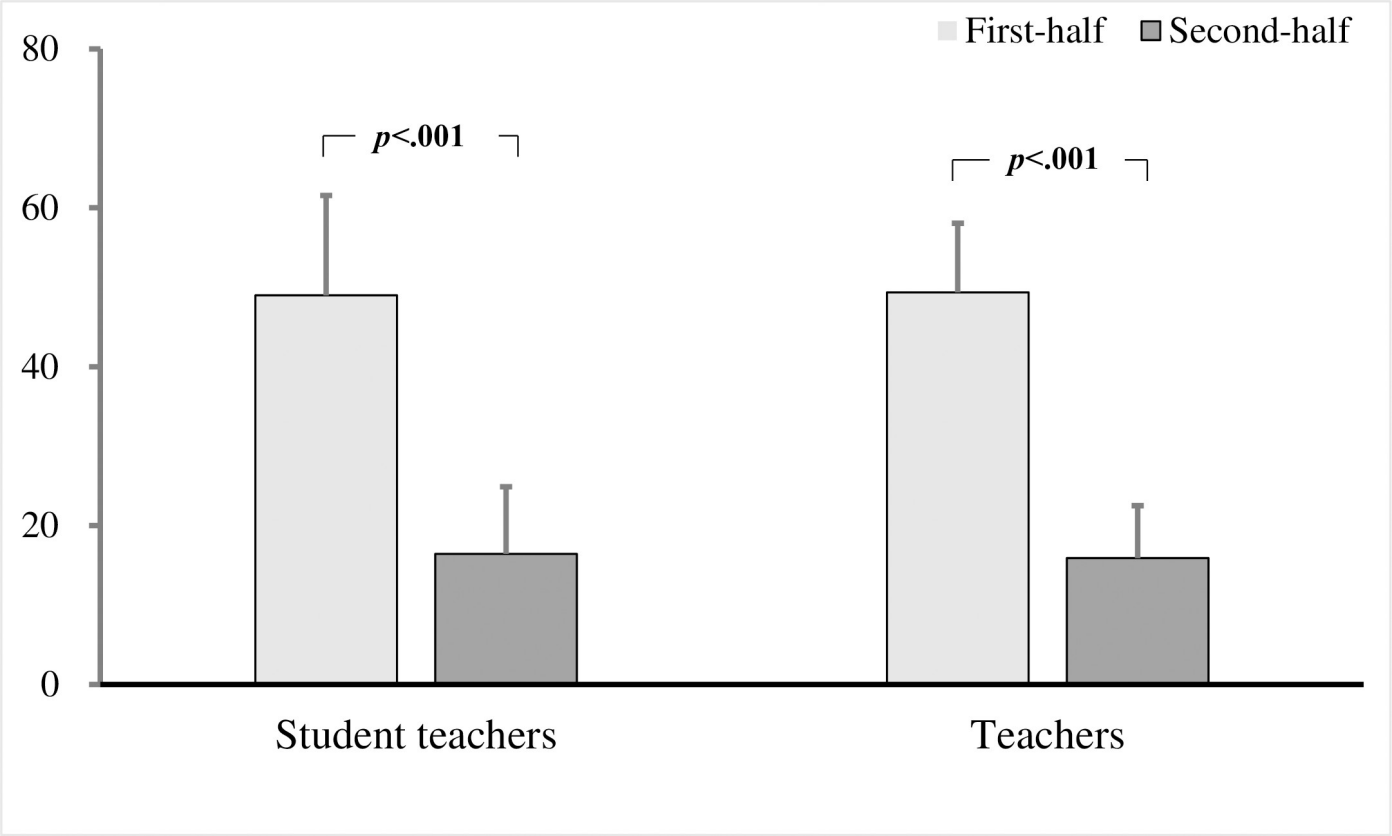
Number of fixations on non-target areas in student teachers and teachers. Bars show the mean across all comparisons (with standard deviation bars).

**Fig 5:**
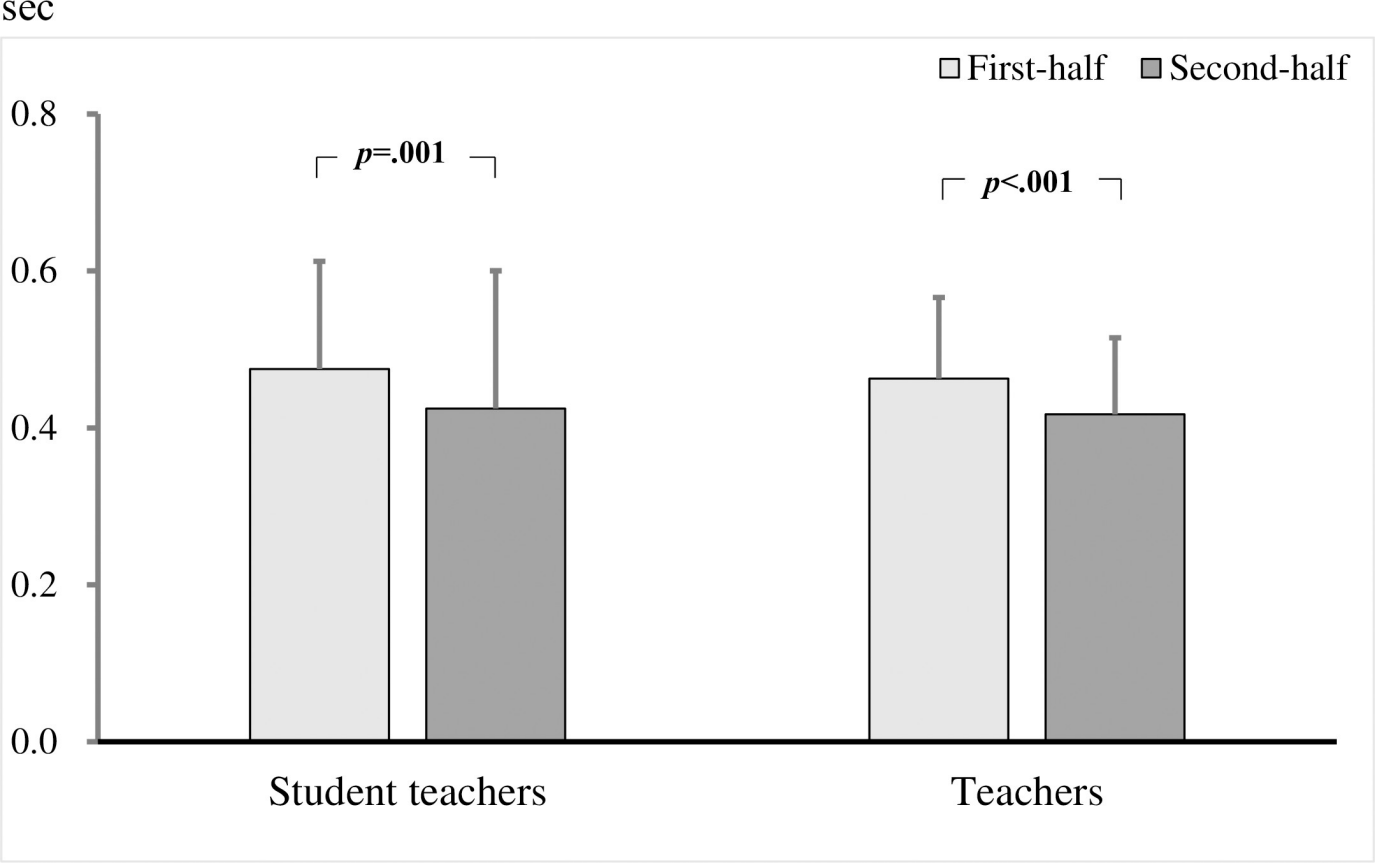
Fixation duration per fixation on non-target areas in student teachers and teachers. Bars show the mean across all comparisons (with standard deviation bars).

To examine differences between teachers and student teachers, a two-factor repeated measures ANOVA was performed for the group (teacher / student) × 2 videos (first half / second half). The interaction effects were not significant in the number of fixations (*F* (1, 221) = .360, *p* = .549), fixation duration (*F* (1, 221) = .013, *p* = .908), and fixation duration per fixation (*F* (1, 221) = .693, *p* = .406; Figs 4 and 5).

### Awareness of students’ off-task behavior

A chi-square test was conducted to determine the difference in teacher and student teachers’ accuracy in noticing the target children (Table 1). As a result, teachers had a higher rate of accurate identification of target children than student teachers did, and the effect size was small (*χ*^2^ = 5.767, *p* = .016, *φ* = 0.161).

**Table 1:** Differences in accuracy in identifying target children between student teachers and teachers.

	Student Teachers	Teachers			
	(*N =* 147)	(*N =* 76)	*χ^2^*	*p*	*φ*
Incorrect	110 (74.8%)	45 (59.2%)	5.767	.016	.161
Correct	37 (25.2%)	31 (40.7%)			

## Discussion

We examined gaze data from 76 teachers and 147 student teachers, which was a larger sample size than in previous eye-tracking studies of teachers. We found that teachers repeatedly gazed toward children showing off-task behavior, more so than student teachers did. We also found that teachers were better at noticing children’s off-task behavior than were student teachers. This indicates that the teachers’ classroom experience has a great influence on their eye movement—that is, visual processing—in response to children’s behavior.

### Difference in gazes of teachers and student teachers

We hypothesized that compared to student teachers, teachers would look more frequently and for longer at children who showed off-task behavior in class because experienced teachers are more efficient in directing their eyes toward the information needed for responsive teaching. In the second half, we found that teachers had more eye fixations on students displaying off-task behaviors in class than student teachers, yet there was no significant difference in the fixation duration between the two groups. Therefore, the increase in fixation duration was correlated with the increase in the number of fixations, and it can be said that the increase in the number of fixations is the difference between the teachers and student teachers. Moreover, there was no significant difference in the number of fixations or fixation duration for the areas other than target children between the two groups. Thus, it seemed that teachers not only gazed at children engaged in off-task behavior but also moved their gaze quickly and frequently, considering other areas of the classroom.

It has been found that the number of fixations increases for places of concern and interest [7]. Based on our findings, it can be inferred that teachers might find it easier to pay attention to children showing off-task behavior than student teachers; that is, the teachers in this study were more aware of such children than the student teachers were. In other words, this study revealed the effectiveness of teachers’ visual processing methods in the classroom. Previous studies provided evidence that experienced teachers are able to see the broader classroom situation functionally, paying attention not only to a child who causes problems but also to how this behavior affects surrounding children [10]. Experienced teachers show a constant and consistent gaze toward a concentrated area, compared to novice instructors [10, 17, 44]. Additionally, McIntyre and Foulsham [45] conducted gaze-tracking research in actual classroom settings and found associations between teacher expertise and quick, frequent gazes toward a child. The results of the current study support the findings of these previous studies by measuring the number of eye fixations and fixation duration on the target children. Furthermore, our findings suggest that experience as a teacher had an effect on a teacher’s frequent gaze toward a specific child.

Additionally, in this study, both student teachers and teachers had a significantly higher number of fixations at the AOI in the second half than in the first half. On the other hand, the number of fixations at the AOI in the second half was significantly higher in teachers than in student teachers. According to a meta-analysis of eye-tracking studies, when developing expertise, individuals optimize processed information by ignoring unnecessary information and actively attending only to relevant information [46]. This is consistent with the information-reduction hypothesis, which asserts that with accumulated experience in a specific area, individuals automatically distinguish between information that is necessary and unnecessary for a task, thus limiting processing to task-related information [47–49]. In this study, teachers with classroom experience gazed at the AOI more frequently than student teachers did. As the AOI was where the target children appeared and it conveyed important information about the classroom, teachers who gazed at the AOI more frequently than student teachers may have recognized the importance of the target children’s behavior within the context of the class situation. We presumed that recognition of the importance of the target children’s behavior led to teachers’ frequent eye fixation on the AOI. This also indicates the influence of experience on teachers’ visual processing in the classroom.

However, the fixation duration in this study was no different between teachers and student teachers. Knowledge of classroom events and classroom situations is built through experience as a teacher, and the accumulated knowledge leads to predictive and conscious responses [43]. Experienced teachers quickly and intensively perceive a particular area of the classroom. From the accumulation of knowledge through past experience, teachers recognized the off-task behavior immediately. Therefore, no further information might have been needed. We also speculated that they were not able to gaze at one place for long because they needed to watch the entire classroom. In the reality of classroom lessons, teachers must be aware of their children’s behavior, repeatedly review and update information, and do so for many children in the classroom. It is presumed that the difference in pedagogical and content knowledge and the difference in experience between teachers and student teachers translated to a difference in the line of sight. This may be a peculiar gaze pattern for teachers who have to look at many students in the class during the limited class time.

The students and classroom in the video used in this study were unfamiliar to the participants. Nevertheless, teaching experience influenced participant gaze toward students who showed off-task behavior (target students). This may depend on differences in schema development. A schema is a knowledge structure that effectively encodes information [50–52]. It is also defined as a framework in which common points are connected hierarchically and become interrelated through the accumulation of experience and practice [50, 53]. In this context, Carter et al. [13] noted that the schemas of novice teachers who faced visual information processing tasks in the classroom were not fully developed. Moreover, the cognitive schemas of novice or student teachers may have reduced elaboration, relevance, and availability, leading to differences in perceptions, interpretations, and thoughts on classroom events, compared to those of experienced teachers [54, 55]. This suggests that the student teachers who participated in this study had not developed schemas for observing children; thus, student teachers could not gaze at the area where teachers gazed quickly and frequently. Conversely, teachers have well-developed schemas for recognizing children’s needs [11, 56, 57]. Additionally, it has also been revealed that the ability to process visual information from large feature patterns is related to the accumulation of experience [58]. Thus, our results suggest that the experience is necessary for teachers who educate many children in one classroom to improve their visual information processing abilities.

Previous studies said that teachers use top-down strategies for visual processing to recognize key information in the classroom and pause or move their gaze to optimal locations to manage the classroom [45, 59]. In the process of developing expertise, individuals come to optimize information by strategic thinking and actively paying attention to relevant information [46]. Based on these findings, experienced teachers might have directed their gaze toward the area where attention should be intensively focused because of their complex schemas. However, because the student teachers did not have such schemas, they were less able to direct their gaze toward meaningful cues and events. Our interpretation is that experience-based differences in schema development influence visual processing, which appeared as a difference in eye-gaze movement toward children with off-task behavior.

### Difference in noticing between teachers and student teachers

Our second hypothesis was that teachers would notice the children who showed off-task behavior in the class more than student teachers would, because teachers select information by preferentially perceiving what is necessary for classroom management [15]. When asked to identify the target children after watching the video, the proportion of teachers who could accurately identify the target children was significantly higher than that of student teachers. This may have been due to the reasons explained below.

As previously mentioned, teachers have an elaborate schema that finds meaning in visual classroom information [13]. Therefore, they have developed the ability to notice and interpret classroom information [57]. Moreover, a well-established schema allows working memory to engage in other activities [60]. Large working memory capacity means great attention usage [61]. Working memory capacity is seen as the ability to control attention to irrelevant information and direct attention to relevant critical information [62, 63]. It involves the distribution of attention to perform the work necessary to keep the information active and quickly searchable [64]. Thus, our results suggest that teachers have a greater working memory capacity than student teachers and use their working memory efficiently by means of a well-established schema, thereby successfully performing their various and complex cognitive activities in the classroom. Furthermore, experienced individuals can better allocate attention to other stimuli and task requirements under practical circumstances [65]. Therefore, the benefits associated with experience may allow teachers to respond to large, complex situations and adapt effectively to exceptional classroom situations [66]. Meanwhile, Sabers, Cushing, and Berliner [67], who studied teachers’ perceptive abilities, found that experienced teachers integrated both visual and auditory stimuli better than novice teachers did. There are also reports that novice teachers overlook the influence and effect teachers have on classroom events when interpreting the context of those events, compared to experienced teachers [9].

These previous findings suggest that the teachers in this study were able to observe various kinds of information in the classroom by using a sufficiently developed schema to notice the students showing off-task behavior in the video. At the same time, presumably, these teachers could also allocate attention to the classroom teacher’s voice when he was warning the students. The thoughts of teachers who have accumulated experience in the field are interpreted as being closely organized with sensory perceptions, such as seeing and hearing, and empirical knowledge [10]. The knowledge accumulated through experience helps observe and understand the interaction between teachers and children [43]. As a result, accumulated educational experience effectively processes audio and visual information. It can be inferred that the teachers could pay attention to the interaction between the classroom teacher and students in class and correctly identify the students who showed off-task behavior in class. Conversely, the student teachers were able to observe the classroom, but overlooked the students who showed off-task behavior in class because their schema was not fully developed. Alternatively, it can be interpreted that the student teachers were unable to notice the off-task behavior because they overlooked the classroom teacher’s warning, although they had their gaze fixed on the students showing off-task behavior in class.

### Limitations

There are several limitations to the current study. First, we compared teachers with student teachers from teacher training colleges, and their ages were different. Most university students (including student teachers) in Japan are under the age of 22, while teachers must be at least 22 years old to obtain a teaching license. Therefore, when comparing teachers and students, there is inevitably a difference in age. It cannot be denied that this difference will affect the results. This has been a limitation in previous studies that compared experienced with beginner teachers or student teachers of differing ages [10, 17, 44]. As age increases, teachers accumulate more experiences in school, which potentially improves their teaching skills as well.

Furthermore, a previous study by Yamamoto and Imai-Matsumura [7] on teachers of different experience levels reported that there was no significant difference in years of teaching experience between teachers who noticed off-task behaviors in class and those who did not. This implies that noticing off-task behaviors can be attributed to natural talent, at least in part. In the current study, we examined whether there was a difference in this skill between in-service teachers who had experience in schools and student teachers who had only practical training and no experience in schools. Our results revealed that the student teachers were inferior at paying attention to off-task children; therefore, we concluded that the ability to notice off-task behaviors is attributed to a teacher’s level of experience as a teacher. It also became clear that this ability could not be acquired by receiving training at a teacher training school. Then, a new research question arose: What kind of teaching experience after becoming a teacher makes a difference in teachers’ ability to notice off-task behaviors? Even with similar years of teaching experience, some teachers are able to notice off-task behaviors in class while others are not [7], suggesting that there may be factors other than years of teaching experience that affect their ability to notice off-task behaviors. Future research could examine differences in the experiences of teachers with considerable and poor ability to identify factors that enable them to notice off-task behaviors. In addition, a longitudinal study of teachers may reveal the changes in their abilities. By doing so, we may be able to find ways to improve the ability of teachers who were not previously capable of noticing such behaviors. Such training could be beneficial for teachers in the future.

A second limitation of the current study is that during the VTR, the teacher made a statement encouraging participation in the class. Because of this statement, it is undeniable that teachers and students who watched the video noticed the child engaging in off-task behavior. However, the classroom teacher in the VTR did not read the name of the target child or specify the child. While observing the entire class, the existence of the target child is not obvious unless the eyes are directed to that child who is engaging in the off-task behavior, which is different from the surroundings. In the future, the effects of auditory stimuli may be clarified by comparing the results with entirely visual stimuli (with no audio), such as the stimuli used in a study by Yamamoto and Imai-Matsumura [7]. It will also be necessary to verify whether the same participants would exhibit differences in the awareness of an off-task behavior when watching a video with both auditory and visual stimuli compared to a video with only visual stimuli.

A third limitation is that we did not compare differences in eye movements between teachers and student teachers in an actual class situation; instead, we used a video with a screen-based eye-tracker. Nevertheless, it is noteworthy that the use of video for improving teaching is becoming common and supports developing the ability to perceive and recognize the cues necessary for learning [68]. Furthermore, research using video is a widely used method for understanding teachers’ expertise and skills [69]. However, only few studies that quantitatively investigate the differences in how gaze is directed between teachers and student teachers using the same class video scene. The significance of this study is that it further clarifies the visual processing of teachers in terms of gazing quickly, frequently, and repeatedly toward children displaying off-task behaviors while also gazing at surrounding areas. However, as this gaze movement was measured while watching a video of a classroom lesson, generalization and application to actual classroom situations must be done with caution. In this study, we examined only the teacher’s gaze on children’s off-task behavior. To clarify the characteristics of visual processing in teachers further, it is necessary to investigate various lesson situations and conduct an in-depth study on how the distribution of gazes differs between teachers and student teachers. In addition, it might be necessary to use mobile eye-trackers at an actual elementary school.

A fourth limitation is that the differences among student teachers in pedagogical and content knowledge and experience are not clarified. We investigated the influence of the presence or absence of experience on gaze after reviewing a study, which concluded that the length of teaching experience did not affect teachers’ gaze [7]. In this study, it was clear that teaching experience did influence teachers’ visual processing. Future studies need to consider differences in experiences and knowledge related to pedagogy held by student teachers. In the primary teacher education system in Japan, it is customary for third-year university students to complete four-week teaching practicums at primary schools. This experience is important for becoming a teacher, and the practical teaching experience may affect visual processing and gaze in student teachers. However, there are no quantitative eye-tracking studies comparing visual processing between students with and without practical teaching experience. Studies that consider the knowledge and experience of student teachers’ pedagogical method and content, especially during the experience of teaching practice, are needed in the future. This information could help clarify the development of visual processing in teachers.

A fifth limitation is that only gaze toward children showing off-task behavior was considered. In previous studies in actual classroom settings, teachers’ gazes were analyzed and categorized as *attentional* or *communicative* [17, 44]. Furthermore, there is research regarding the fairness ratio by calculating the Gini coefficient to consider how evenly a teacher observes children [18]. To clarify the characteristics of teachers’ visual processing further, it will be necessary to conduct empirical research on the gaze of teachers from various perspectives.

### Implications

For effective teaching, it is important to pay attention to students’ off-task behaviors in class. However, focusing one’s gaze on a particular student can lead to neglect of the other students in the classroom. Therefore, teachers need to look at the students engaging in off-task behaviors quickly and frequently, while keeping an eye on the other students as well. It would also be beneficial for student teachers, who are about to become teachers, to learn where teachers gaze most often and how they position their gaze. This would help student teachers develop their ability to observe all the students in a class. In this context, recorded models of experienced teachers’ gaze can be used to help student teachers learn how to allocate visual attention effectively [16]. Thus, teachers’ knowledge of visual processing through direct measures of gaze can contribute significantly to teacher training.

## Supporting information

S1 Data (XLSX)

## References

[1] Eilam B., & Poyas Y. (2006). Promoting awareness of the characteristics of classrooms’ complexity: A course curriculum in teacher education. *Teaching and Teacher Education*, 22(3), 337–351. 10.1016/j.tate.2005.11.004.

[2] van Es E. A., & Sherin M. G. (2002). Learning to notice: Scaffolding new teachers’ interpretations of classroom interactions. *Journal of Technology and Teacher Education*, 10(4), 571–596.

[3] Lin X., Schwartz D. L., & Hatano G. (2005). Toward teachers’ adaptive metacognition. *Educational Psychologist*, 40(4), 245–255.

[4] Seidel T., & Stürmer K. (2014). Modeling and measuring the structure of professional vision in preservice teachers. *American Educational Research Journal*, 51(4), 739–771. 10.3102/0002831214531321.

[5] Yoon S. A., Koehler-Yom J., Anderson E., Lin J., & Klopfer E. (2015). Using an adaptive expertise lens to understand the quality of teachers’ classroom implementation of computer-supported complex systems curricula in high school science. *Research in Science & Technological Education*, 33(2), 237–251. 10.1080/02635143.2015.1031099

[6] Lobman C. L. (2006). Improvisation: An analytic tool for examining teacher–child interactions in the early childhood classroom. *Early Childhood Research Quarterly*, 21(4), 455–470. 10.1016/j.ecresq.2006.09.004.

[7] Yamamoto T., & Imai-Matsumura K. (2013). Teachers’ gaze and awareness of students’ behavior: Using an eye tracker. *Comprehensive Psychology*, 2. 10.2466/01.IT.2.6.

[8] Stürmer K., Könings K. D., & Seidel T. (2013). Declarative knowledge and professional vision in teacher education: Effect of courses in teaching and learning. *British Journal of Educational Psychology*, 83(3), 467–483. doi: 10.1111/j.2044-8279.2012.02075.x 23822532

[9] Wolff C. E., Jarodzka H., & Boshuizen H. P. A. (2017). See and tell: Differences between expert and novice teachers’ interpretations of problematic classroom management events. *Teaching and Teacher Education*, 66, 295–308. 10.1016/j.tate.2017.04.015.

[10] Wolff C. E., Jarodzka H., van den Bogert N., & Boshuizen H. P. A. (2016). Teacher vision: Expert and novice teachers’ perception of problematic classroom management scenes. *Instructional Science*, 44(3), 243–265. 10.1007/s11251-016-9367-z.

[11] Westerman D. A. (1991). Expert and novice teacher decision making. *Journal of Teacher Education*, 42(4), 292–305.

[12] Fogarty, J. L., Wang, M. C., & Creek, R. (1982). A descriptive study of experienced and novice teachers’ interactive instructional decision processes. *Paper presented at the annual meeting of the American Educational Research Association*, New York City.

[13] Carter K., Cushing K., Sabers D., Stein P., & Berliner D. (1988). Expert-novice differences in perceiving and processing visual classroom information. *Journal of Teacher Education*, 39(3), 25–31.

[14] Swanson H. L., O’Connor J. E., & Cooney J. B. (1990). An information processing analysis of expert and novice teachers’ problem solving. *American Educational Research Journal*, 27(3), 533–556.

[15] Berliner D. C. (2001). Learning about and learning from expert teachers. *International Journal of Educational Research*, 35, 463–482.

[16] van den Bogert N., van Bruggen J., Kostons D., & Jochems W. (2014). First steps into understanding teachers’ visual perception of classroom events. *Teaching and Teacher Education*, 37, 208–216. 10.1016/j.tate.2013.09.001.

[17] McIntyre N. A., Mainhard M. T., & Klassen R. M. (2017). Are you looking to teach? Cultural, temporal and dynamic insights into expert teacher gaze. *Learning and Instruction*, 49, 41–53. 10.1016/j.learninstruc.2016.12.005.

[18] Cortina K. S., Miller K. F., McKenzie R., & Epstein A. (2015). Where low and high inference data converge: Validation of CLASS assessment of mathematics instruction using mobile eye tracking with expert and novice teachers. *International Journal of Science and Mathematics Education*, 13(2), 389–403. 10.1007/s10763-014-9610-5.

[19] Poole A., & Ball L. (2006). Eye tracking in human-computer interaction and usability research. Current status and future prospects. In: Ghaoui C., editor. Encyclopedia of human computer interaction. Hershey,PA: Idea Group; 2006. p. 211–219.

[20] Meltzoff A. N., Brooks R., Shon A. P., & Rao R. P. N. (2010). “Social” robots are psychological agents for infants: A test of gaze following. Neural Networks, 23, 966–972. doi: 10.1016/j.neunet.2010.09.005 20951333 PMC7089732

[21] Goldberg J., & Helfman J. (2011). Eye tracking for visualization evaluation: Reading values on linear versus radial graphs. Information Visualization, 10(3), 182–195. doi: 10.1177/1473871611406623

[22] Liversedge S. P., & Findlay J. M. (2000). Saccadic eye movements and cognition. Trends in Cognitive Sciences, 4(1), 6–14. doi: 10.1016/s1364-6613(99)01418-7 10637617

[23] van Gog T., & Scheiter K. (2010). Eye tracking as a tool to study and enhance multimedia learning. *Learning and Instruction*, 20(2), 95–99. doi: 10.1016/j.learninstruc.2009.02.009

[24] Kasarskis, P., Stehwien, J., Hickox, J., Aretz, A., & Wickens, C. (2001). Comparison of expert and novice scan behaviors during VFR flight. In *Paper presented at the 11th International Symposium on Aviation Psychology, Columbus, OH*.

[25] Bednarik R. (2012). Expertise-dependent visual attention strategies develop over time during debugging with multiple code representations. *International Journal of Human-Computer Studies*, 70(2), 143–155. 10.1016/j.ijhcs.2011.09.003.

[26] Charness N., Reingold E. M., Pomplun M., & Stampe D. M. (2001). The perceptual aspect of skilled performance in chess: Evidence from eye movements. Memory & Co*gnition*, 29(8), 1146–1152. doi: 10.3758/bf03206384 11913751

[27] Sheridan H., & Reingold E. M. (2014). Expert vs. novice differences in the detection of relevant information during a chess game: evidence from eye movements. *Frontiers in Psychology*, 5, 941. 10.3389/fpsyg.2014.00941. 10.3389/fpsyg.2014.00941. 25202298 PMC4142462

[28] Savelsbergh G. J. P., Williams A. M., van der Kamp J., & Ward P. (2002). Visual search, anticipation and expertise in soccer goalkeepers. *Journal of Sports Sciences*, 20(3), 279–287. doi: 10.1080/026404102317284826 11999482

[29] Spitz J., Put K., Wagemans J., Williams A. M., & Helsen W. F. (2016). Visual search behaviors of association football referees during assessment of foul play situations. *Cognitive Research*: *Principles and Implications*, 1, 12. 10.1186/s41235-016-0013-8. PMC5256438 28180163

[30] Roca A., Ford P. R., McRobert A. P., & Williams A. M. (2011). Identifying the processes underpinning anticipation and decision-making in a dynamic time-constrained task. *Cognitive Processing*, 12(3), 301–310. doi: 10.1007/s10339-011-0392-1 21305386

[31] Koide N., Kubo T., Nishida S., Shibata T., & Ikeda K. (2015). Art expertise reduces influence of visual salience on fixation in viewing abstract-paintings. *PLoS One*, 10(2), e0117696. doi: 10.1371/journal.pone.0117696 25658327 PMC4319974

[32] Brunye T. T., Carney P. A., Allison K. H., Shapiro L. G., Weaver D. L., & Elmore J. G. (2014). Eye movements as an index of pathologist visual expertise: A pilot study. *PLoS One*, 9(8), e103447. doi: 10.1371/journal.pone.0103447 25084012 PMC4118873

[33] Jarodzka H., Scheiter K., Gerjets P., & van Gog T. (2010). In the eyes of the beholder: How experts and novices interpret dynamic stimuli. *Learning and Instruction*, 20(2), 146–154. 10.1016/j.learninstruc.2009.02.019.

[34] Krupinski E. A., Tillack A. A., Richter L., Henderson J. T., Bhattacharyya A. K., Scott K. M.,… et al. (2006). Eye-movement study and human performance using telepathology virtual slides: Implications for medical education and differences with experience. *Human Pathology*, 37(12), 1543–1556. doi: 10.1016/j.humpath.2006.08.024 17129792

[35] Andrà, C., Arzarello, F., Ferrara, F., Holmqvist, K., Lindström, P., Robutti, O., et al. (2009). How students read mathematical representations: An eye tracking study. *Proceedings of the 33rd Conference of the International Group for the psychology of mathematics education, 2*, 49–56.

[36] Inglis M., & Alcock L. (2012). Expert and novice approaches to reading mathematical proofs. *Journal for Research in Mathematics Education*, 43(4), 358–390. https://www.jstor.org/stable/10.5951/jresematheduc.43.4.0358.

[37] Hutton SB and Nolte S (2011) The effect of gaze cues on attention to print advertisements. Applied Cognitive Psychology 25(6): 887–892.

[38] Uzzaman S., & Joordens S. (2011). The eyes know what you are thinking: Eye movements as an objective measure of mind wandering. Consciousness and Cognition, 20, 1882–1886. doi: 10.1016/j.concog.2011.09.010 21968150

[39] Fisher C., Berliner D., Filby N., Marliave R., Cahen L., & Dishaw M. (1981). Teaching behaviours, academic learning time, and student achievement: an overview. *The Journal of Classroom Interactions*, 17(1), 2–15.

[40] Karweit N., & Slavin R. E. (1981). Measurement and modeling choices in studies of time and learning. *American Educational Research Journal*, 18(2), 157–171.

[41] Lee S. W., Kelly K. E., & Nyre J. E. (1999). Preliminary report on the relation of students’ on-task behavior with completion of school work. *Psychological Reports*, 84(1), 267–272.

[42] Beach P., & McConnel J. (2019). Eye tracking methodology for studying teacher learning: A review of the research. International Journal of Research & Me*thod in Education*, 42(5), 485–501. 10.1080/1743727X.2018.1496415

[43] Wolff C. E., Jarodzka H., & Boshuizen H. P. A. (2020). Classroom management scripts:A theoretical model contrasting expert and novice teachers’ knowledge andawareness of classroom events. *Educational Psychology Review*. 10.1007/s10648-020-09542-0

[44] McIntyre N. A., Jarodzka H., & Klassen R. M. (2017). Capturing teacher priorities: Using real-world eye-tracking to investigate expert teacher priorities across two cultures. *Learning and Instruction*. doi: 10.1016/j.learninstruc.2017.03.005 29051690 PMC5642925

[45] McIntyre N. A., & Foulsham T. (2018). Scanpath analysis of expertise and culture in teacher gaze in real-world classrooms. *Instructional Science*, 46(3), 435–455. 10.1007/s11251-017-9445-x.

[46] Gegenfurtner A., Lehtinen E., & Säljö R. (2011). Expertise differences in the comprehension of visualizations: a meta-analysis of eye-tracking research in professional domains. *Educational Psychology Review*, 23(4), 523–552. 10.1007/s10648-011-9174-7.

[47] Haider H., & Frensch P. A. (1996). The role of information reduction in skill acquisition. *Cognitive Psychology*, 30(3), 304–337. doi: 10.1006/cogp.1996.0009 8660787

[48] Haider H., & Frensch P. A. (1999a). Eye movement during skill acquisition: More evidence for the information-reduction hypothesis. *Journal of Experimental Psychology*: *Learning*, *Memory*, *and Cognition*, 25(1), 172–190.

[49] Haider H., & Frensch P. A. (1999b). Information reduction during skill acquisition: The influence of task instruction. *journal of* *Experimental Psychology*, 5(2), 129–151.

[50] Ghosh V. E., & Gilboa A. (2014). What is a memory schema? A historical perspective on current neuroscience literature. *Neuropsychologia*, 53, 104–114. doi: 10.1016/j.neuropsychologia.2013.11.010 24280650

[51] Posner M. I., & Keele S. W. (1968). On the genesis of abstract ideas. *Journal of Experimental Psychology*, 77(3), 353–363. doi: 10.1037/h0025953 5665566

[52] Rest J., Narvaez D., Bebeau M., & Thoma S. (1999). A neo-Kohlbergian approach: The DIT and schema theory. *Educational Psychology Review*, 11(4), 291–324.

[53] Anderson R. C. (1984). Some reflections on the acquisition of knowledge. *Educational Researcher*, 13(9), 5–10.

[54] Borko H., & Livingston C. (1989). Cognition and improvisation: Differences in mathematics instruction by expert and novice teachers. *American Educational Research Journal*, 26(4), 473–498. 10.3102/00028312026004473.

[55] Livingston C., & Borko H. (1990). High school mathematics review lessons: Expert-novice distinctions. *Journal for Research in Mathematics Education*, 21(5), 372–387.

[56] Calderhead, J. (1983). Research into teachers’ and student teachers’ cognitions: Exploring the nature of classroom practice. Paper presented at the annual meeting of the American Educational Research Association, Montreal, Canada.

[57] Carter K., Sabers D., Cushing K., Pinnegar S., & Berliner D. C. (1987). Processing and using information about students: A study of expert, novice, and postulant teachers. *Teaching and Teacher Education*, 3(2), 147–157.

[58] Reingold E. M., & Sheridan H. (2011). Eye movements and visual expertise in chess and medicine. In Liversedge S. P, Gilchrist I. D, & Everling S. (Eds.), *Oxford handbook on eye movements* (pp. 767–786). Oxford, UK: Oxford University Press.

[59] Kim L. E., & Klassen R. M. (2018). Teachers’ cognitive processing of complex school-based scenarios: Differences across experience levels. *Teaching and Teacher Education*, 73, 215–226. 10.1016/j.tate.2018.04.006.

[60] Sweller J., van Merriënboer J. J. G., & Paas F. G. W. C. (1998). Cognitive architecture and instructional design. *Educational Psychology Review*, 10(3), 251–296.

[61] Engle R. W. (2002). Working memory capacity as executive attention. *Current Directions in Psychological Science*,11, 19–23. doi: 10.3758/bf03196323 12613671

[62] Engle R. W., & Kane M. J. (2004). Executive attention, working memory capacity, and a two-factor theory of cognitive control. In Ross B (Ed.), *The psychology of learning and motivation* (Vol. 44, pp. 145–199). New York: Elsevier.

[63] Shipstead Z., Lindsey D. R. B., Marshall R. L., & Engle R. E. (2014). The mechanisms of working memory capacity: Primary memory, secondary memory, and attention control. *Journal of Memory and Language*, 72, 116–141. 10.1016/j.jml.2014.01.004

[64] Bleckley M. K., Foster J. L., & Engle R. W. (2015). Working memory capacity accounts for the ability to switch between object-based and location-based allocation of visual attention. *Memory & Cognition*, 43, 379–388. doi: 10.3758/s13421-014-0485-z 25421317

[65] Beilock S.L., Wierenga S.A., & Carr T.H. (2002). Expertise, attention, and memory in sensorimotor skill execution: Impact of novel task constraints on dual-task performance and episodic memory. *Quarterly Journal of Experimental Psychology Section A* 55(4), 1211–1240. 10.1080/02724980244000170. 12420993

[66] Feldon D. F. (2007). Cognitive load and classroom teaching: The double-edged sword of automaticity. *Educational Psychologist*, 42(3), 123–137. 10.1080/00461520701416173.

[67] Sabers D. S., Cushing K. S., & Berliner D. C. (1991). Differences among teachers in a task characterized by simultaneity, multidimensional, and immediacy. *American Educational Research Journal*, 28(1), 63–88. 10.3102/00028312028001063.

[68] Gold B., & Holodynski M. (2017). Using digital video to measure the professional vision of elementary classroom management: Test validation and methodological challenges. Computers & Education, 107, 13–30. 10.1016/j.compedu.2016.12.012.

[69] Gaudin C., & Chaliès S. (2015). Video viewing in teacher education and professional development: A literature review. *Educational Research Review*, 16, 41–67. 10.1016/j.edurev.2015.06.001.

